# Large-Scale Dissemination of Internet-Based Cognitive Behavioral Therapy for Youth Anxiety: Feasibility and Acceptability Study

**DOI:** 10.2196/jmir.9211

**Published:** 2018-07-04

**Authors:** Sonja March, Susan H Spence, Caroline L Donovan, Justin A Kenardy

**Affiliations:** ^1^ Institute for Resilient Regions University of Southern Queensland Springfield, QLD Australia; ^2^ School of Psychology and Counselling University of Southern Queensland Ipswich, QLD Australia; ^3^ Australian Institute for Suicide Research and Prevention Griffith University Mt Gravatt, QLD Australia; ^4^ School of Applied Psychology Griffith University Mt Gravatt, QLD Australia; ^5^ School of Psychology University of Queensland St Lucia, QLD Australia

**Keywords:** adolescent, child, anxiety disorders, cognitive behavioral therapy, eHealth, public health

## Abstract

**Background:**

Internet-based cognitive behavioral therapy (iCBT) for child and adolescent anxiety has demonstrated efficacy in randomized controlled trials, but it has not yet been examined when disseminated as a public health intervention. If effective, iCBT programs could be a promising first-step, low-intensity intervention that can be easily accessed by young people.

**Objective:**

The objective of our study was to examine the feasibility and acceptability of a publicly available online, self-help iCBT program (BRAVE Self-Help) through exploration of program adherence, satisfaction, and changes in anxiety.

**Methods:**

This study was an open trial involving the analysis of data collected from 4425 children and adolescents aged 7-17 years who presented with elevated anxiety at registration (baseline) for the iCBT program that was delivered through an open-access portal with no professional support. We assessed the program satisfaction via a satisfaction scale and measured adherence via the number of completed sessions. In addition, anxiety severity was assessed via scores on the Children’s Anxiety Scale, 8-item (CAS-8) at four time points: baseline, Session 4, Session 7, and Session 10.

**Results:**

Participants reported moderate satisfaction with the program and 30% completed three or more sessions. Statistically significant reductions in anxiety were evident across all time points for both children and adolescents. For users who completed six or more sessions, there was an average 4-point improvement in CAS-8 scores (Cohen *d*=0.87, children; Cohen *d*=0.81, adolescents), indicating a moderate to large effect size. Among participants who completed nine sessions, 57.7% (94/163) achieved recovery into nonelevated levels of anxiety and 54.6% (89/163) achieved statistically reliable reductions in anxiety.

**Conclusions:**

Participant feedback was positive, and the program was acceptable to most young people. Furthermore, significant and meaningful reductions in anxiety symptoms were achieved by many children and adolescents participating in this completely open-access and self-directed iCBT program. Our results suggest that online self-help CBT may offer a feasible and acceptable first step for service delivery to children and adolescents with anxiety.

## Introduction

Half of all lifetime mental health disorders begin before the age of 14 years [1], highlighting the importance of early intervention as a strategy for promoting lifelong mental health. Anxiety is one of the most common childhood mental health conditions, with almost 7% of Australian children and adolescents aged 4-17 years meeting the criteria for an anxiety disorder [2,3]. Although anxiety disorders in youth lead to significant impairment [4], they can be treated effectively using cognitive behavioral therapy (CBT) [5]. Unfortunately, only 56% of young people with mental disorders report having used services in the previous 12 months, with only 2.2% accessing specialist child and adolescent mental health care [2]. The pervasiveness of anxiety and the noted barriers to treatment [6,7] highlight the importance and potential value of evidence-based, population-level early interventions.

The current Australian federal government recommendations encourage primary prevention and early intervention across the life span through easy-to-access first-line responses, particularly for children [8]. They further propose the use of digital and low-intensity mental health services to ensure that all Australians have access to care, before crisis, irrespective of their geographical location [8].

BRAVE Self-Help is an online, open-access, self-help intervention for child and adolescent anxiety that addresses the needs of anxious Australian youth using a digital, low-intensity, and population-level model. The BRAVE Self-Help initiative was initially supported by *beyondblue* and commenced in 2014, offering an evidence-based, open-access, online program free of charge to Australian young people and their parents. The self-help program was adapted from BRAVE-ONLINE, a 10-session, internet-based CBT (iCBT) program implemented with brief therapist support. The evidence base for the therapist-assisted program is strong [9-12], and the program is recognized internationally as the only “probably efficacious” iCBT intervention for childhood anxiety [13]. Furthermore, the program assists young people to develop strategies for identifying and managing anxiety-provoking situations using youth-friendly, engaging, and interactive Web-based sessions. The objective of the current initiative was to examine its feasibility and acceptability when disseminated nationally and offered as an open-access, self-help, early intervention program for young Australians without therapist support.

There has been relatively little research examining the implementation of iCBT programs for child and adolescent anxiety in real-world clinical and community contexts (eg, outside university-based research trials); furthermore, there are no formalized guidelines for a large-scale dissemination [14]. One small feasibility study has very recently been reported by Jolstedt et al [15], where an evidence-based iCBT program for anxiety was implemented in a small sample (N=20) of anxious children in an outpatient clinic in Sweden. The program was delivered with therapist support (20 min/week) and included both child and parent involvement. Overall, the program was acceptable (moderate to high satisfaction) to young people, parents, and clinicians, with participants completing, on average, 6 out of 12 modules and half of the sample reaching at least module 4 (exposure). Furthermore, young people showed significant reductions in anxiety symptoms from pre- to posttreatment (Cohen *d*=1.22). Thus, the study by Jolstedt et al provided some preliminary support for the dissemination of iCBT interventions in real-world settings, although it was limited to a specific outpatient setting rather than national and open dissemination and participants were required to meet strict inclusion criteria. Thus, further research is required to understand the feasibility of disseminating iCBT programs to large groups of children and adolescents.

Despite substantial challenges involved in determining the outcomes of an open-access, real-world service, we examined the impact of the BRAVE Self-Help intervention through a feasibility and acceptability approach. As the program was developed and intended for youth with anxiety, we were specifically interested in those children and adolescents who reported elevated levels of anxiety at enrollment into the self-help program. Our primary aim was to determine the feasibility and acceptability of BRAVE Self-Help when disseminated nationally through open access. Specifically, we evaluated the level of adherence to and satisfaction with the program as well as the extent to which anxiety symptoms changed over the course of the program for those with elevated symptoms. We hypothesized that children and adolescents who adhered to the program would show significant reductions in their self-reported anxiety from baseline to Sessions 4, 7, and 10. We also expected high satisfaction among participants of the program.

## Methods

### Participants and Procedure

Participants were 4425 anxious young people aged 7-17 years (1473 children aged 7-12 years; 2952 adolescents aged 13-17 years) with mean (SD) age 12.95 (2.97) years; there were 66.39% (2938/4425) females and 31.77% (1406/4425) males, and 1.84% (81/4425) participants identified as another gender category. In terms of residence, 57.45% (2542/4425) participants resided in major cities, with 23.35% (1033/4425) from Inner Regional Australia, 11.21% (496/4425) from Outer Regional Australia, and 2.55% (113/4425) living in remote or very remote Australia (241/4425 [5.44%] provided data that could not be accurately coded).

All participants registered for the program through a website accessible only to Australian families. Then, participants were directed to the program in several ways through (1) self-referral and internet searching, (2) referral from health or education professionals, (3) links hosted on several Australian mental health information sites (eg, Reach Out and Beacon), and (4) direct links from the *beyondblue* and *youthbeyondblue* websites. Through the *beyondblue* website, the BRAVE Program was listed as a direct referral for young people who completed an anxiety quiz and scored high on anxiety. In order to promote awareness of the program, introduction letters, flyers, and postcards were sent to schools and health and mental health organizations as well as private practitioners nationwide. The program was also presented at relevant conferences held for school counselors, psychologists, and teachers. Throughout the 2-year recruitment period, 28.67% (1269/4425) of participants were referred by school-based professionals, 13.36% (591/4425) by external health professionals, 10.85% (480/4425) by a parent or family member, 8.48% (375/4425) through *beyondblue*, and 9.94% (440/4425) through internet searching, with the remaining (28.70%, 1270/4425) participants finding the program through other means (eg, word-of-mouth, radio, magazine, and advertisements). With respect to the participants referred from health professionals, 13.96% (80/573) were referred by their general practitioner, 55.67% (319/573) by a psychologist, and 9.60% (55/573) by a social worker, with the remaining participants referred by other health professionals.

**Figure 1:**
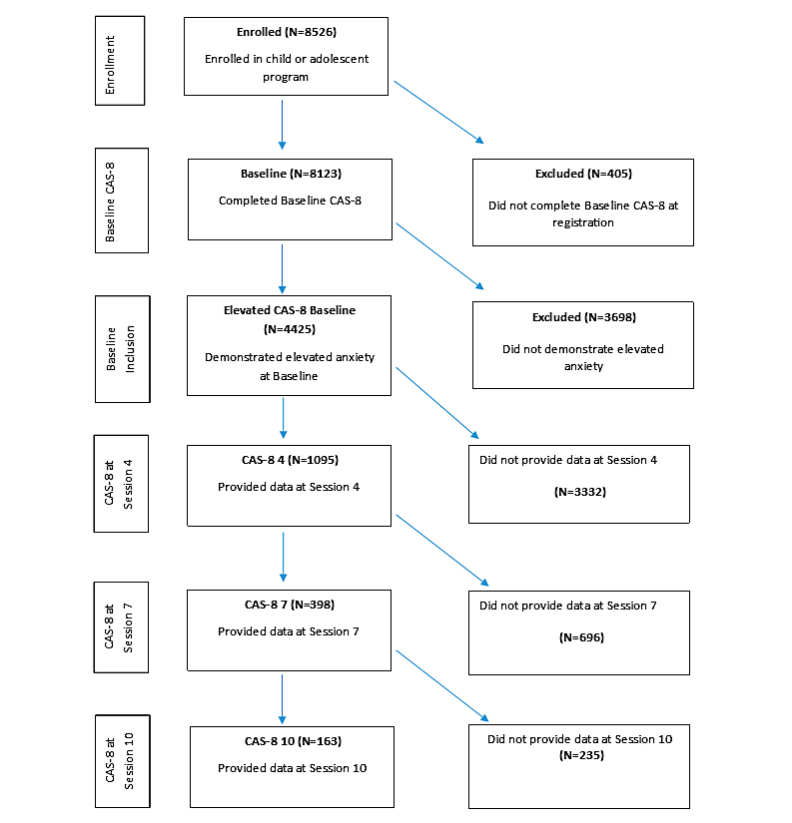
The progression of participants through the program. CAS-8: Children’s Anxiety Scale, 8-item.

To be included in this study, participants were required to have enrolled in the BRAVE Self-Help program between July 1, 2014, and June 30, 2016. We monitored the program progress for participants through to November 17, 2016. All participants (including those registered at the end of the recruitment period) were monitored over a 20-week period, which was sufficient to complete the ten sessions. Participants who were enrolled outside of the 2-year recruitment period were excluded from this study. As this online program is open-access, young people do not need a referral to register and begin the sessions. Besides, there are no set inclusion criteria for enrollment in and access to the program, and thus, users are able to access the program for prevention, early intervention, or treatment purposes. When registering, users are not required to demonstrate symptomatic levels of anxiety, and the program is completely open access and self-sought. In this study, however, participants were included only if they demonstrated elevated anxiety at the baseline above a predetermined criterion (≥84th percentile or *T*-score ≥60 on the Children’s Anxiety Scale [CAS-8]; see below). The progression of participants from registration through the study, with reasons for exclusion is presented in Figure 1.

There are two versions of the program, one for children aged 7-12 years and another for teenagers aged 13-17 years; participants selected which version they wish to complete. Informed consent (and parental consent in the case of children aged <16 years) was required prior to beginning the program, and it was obtained during the Web-based registration process. Participation was voluntary, and young people were made aware that they could cease using the program at any time, without consequence. The study protocol approval was obtained from the ethics committees of the University of Queensland (UQ), University of Southern Queensland, and Griffith University. Furthermore, data were stored on secure servers hosted by UQ. Participants were not provided with any reimbursements for participation.

### Clinical Intervention

The BRAVE-ONLINE program for youth anxiety when delivered with minimal therapist assistance has been described elsewhere [10-12,16]. For this study, the program content (modules) remained the same as for the therapist-assisted program (including 10 interactive Web-based CBT sessions), although minor adaptations were made to the presentation of material to facilitate the learning and implementation of the complex CBT strategies in the absence of a therapist. Volunteer young people from the target age groups were included in the development process, providing feedback on the look, feel, and functionality of the key added components (eg, relaxation room and exposure hierarchy tool) through two iterations. In addition, an expert advisory panel comprising the research team, two expert advisors, stakeholder representatives, and two youth advisors provided feedback throughout the development and delivery process.

In addition to the removal of any therapist contact, the following changes were made to the existing intervention. First, we created a new infrastructure to surround the existing program and provide clearer navigation to resources for users. This included additional home pages, new graphics, demonstration videos, frequently asked questions, and dedicated sections for resources and key program components (eg, relaxation room and exposure section). Second, we integrated relaxation activities into the program via the dedicated relaxation room, where users could live stream relaxation or download relaxation recordings (or transcripts) to add to their music libraries. Third, the exposure hierarchy (BRAVE ladder) was integrated into the new home page infrastructure in a dedicated section and additional tools were created to ensure that young participants were able to build their exposure hierarchy effectively without a therapist. This included easy creation of steps and rewards, the ability to move steps around, and the capacity to check-off steps when complete. Fourth, in addition to the already included automatic email reminders and session completion messages, the self-help program also included automatic alert messages that were sent to the users if they reported anxiety scores in the clinical range. Finally, we integrated a self-registration system into the program that required young people to register for the program and provide a contact email address and name or pseudonym. Registration also included the provision of consent and an explanation of the monitoring and reporting features included in the program. All users were able to first trial the program in a 20-minute guest access before registering. Young people were able to register for and complete the program on their own, via a personal computer or mobile device. Program sessions were conducted in a prescribed sequence, although participants could progress at their own pace. Furthermore, automatic email reminders were sent if participants had not completed the next session within 1 week.

### Measures

In this study, we aimed to disseminate the intervention as widely as possible on a national scale; thus, we implemented assessment procedures conducive to this goal. The advisory committee identified administering an exhaustive assessment battery as a potential deterrent for young people registering and completing the program in this self-directed manner. Thus, to minimize participant burden and the potential barriers to participation, we administered a limited assessment battery, including one brief measure of anxiety, along with basic demographic characteristics and a brief satisfaction scale.

#### Demographics

Demographic data (eg, age, gender, and postcode) were collected when participants created their account to access the program. Postcodes were categorized according to the Australian Standard Geographic Classification system [17] and coded into Major City, Inner Regional, Outer Regional, Remote, and Very Remote. For the purpose of categorical analysis, these categories were coded into Major Cities and Outside Major Cities.

#### Anxiety

We measured anxiety symptom severity using CAS-8 [18], an 8-item scale adapted from the Spence Children’s Anxiety Scale [19], assessing child anxiety symptoms on a 4-point scale (0=*Never*, 3=*Always*). The CAS-8 has demonstrated good reliability and provides population-level, gender-standardized norms for comparison [18]. Scores of ≥84th percentile (ie, above a *T*-score of 60: CAS-8 score ≥10 for males and ≥12 for females) are considered indicative of elevated anxiety, while scores of ≥94th percentile (ie, above a *T*-score of 65: CAS-8 score ≥13 for males and ≥16 for females) are considered representative of clinical levels of anxiety. In this study, participants completed the CAS-8 prior to beginning the program (baseline) and at the beginning of Sessions 4, 7, and 10 (ie, after completion of Sessions 3, 6, and 9). In addition, the CAS-8 was integrated into the program such that it was conducted before the participant could progress with the session. The internal consistency of the CAS-8 for data collected throughout this program was 0.85.

#### Adherence and Satisfaction

Session completion (adherence) was operationalized as the number of program sessions completed and was automatically recorded by the program. We measured both satisfaction and acceptability via a 5-item scale based on a satisfaction questionnaire administered in previous trials of the BRAVE Program [10,11]. Satisfaction data were measured at the same time points as the CAS-8 and were examined based on the responses to the final (latest) satisfaction assessment completed by each participant. In addition, participants were required to respond to items assessing whether they would tell a friend about the program (Item 1), how helpful the program was (Item 2), how happy they were with the program (Item 3), how much the program helped to reduce their anxiety (Item 4), and overall judgment of the program (Item 5). Responses to the 5 items were provided on a 5-point Likert scale, with responses for item 1 scored as 1=*Definitely Not*, 3=*Maybe*, and 5=*Definitely Yes*; responses for items 2, 3, and 4 scored as 1=*Not at all*, 3=*Quite a bit*, and 5=*Very Much*; and responses for item 5 scored as 1=*Very Bad*, 3=*Okay*, and 5=*Very Good*. We calculated the mean item and mean total satisfaction scores. An additional final item was included as a free-text, qualitative item asking the participant to comment on anything else about the program. A sample of responses to this question is provided in the Results.

#### Safety Alerts

Given the self-help nature of this intervention, the program was designed to incorporate checks on anxiety, alerts regarding participants who were experiencing high levels of anxiety, and the provision of appropriate referral information. In this study, any child or adolescent scoring in the clinical ranges of anxiety at any assessment point was sent an automatic message that alerted the person to his or her high score and encouraged him or her to seek further support from additional sources, including family, friends, and professional services. The message also included contact details for crisis lines and services. We calculated the proportion of children and adolescents receiving email alerts at the four different assessment points.

### Statistical Analysis

We used IBM Statistics 24 and MPlus 8 for statistical analyses. Descriptive data for satisfaction item and total means were evaluated and presented for the total sample as well as child and adolescent subsamples. Examples of feedback are provided as well. Furthermore, descriptive data for program adherence (number of sessions completed) were evaluated and reported for the total sample as well as according to child and adolescent samples. In terms of safety alerts, the proportion of participants receiving email alerts at each time point was calculated. Baseline differences in anxiety severity, age, gender, and geographic location between the participants who completed less than three sessions and those who completed three or more sessions were examined using *t* tests and chi-square tests. Furthermore, the relationship between program compliance and CAS-8 scores at the final program attendance was expressed as a Pearson product-moment correlation coefficient (*r*). Change in anxiety was analyzed in three ways following recommendations for determining therapeutic changes in child and adolescent populations [20]. Given the lack of a control comparison condition, providing multiple inferences of the data through different means allowed checks for common patterns in results and increased confidence in the results observed.

First, we examined the mean change in raw anxiety scores across the program. Following the procedure of Rickwood et al [21] in their implementation evaluation of the headspace service, analyses were first conducted with treatment “completers,” ie, those who provided data at the relevant time points. Therefore, to determine changes in anxiety scores from baseline to Session 4, baseline to Session 7, and baseline to Session 10, separate repeated-measures analyses of variance (ANOVA) were conducted with individuals who had completed the sessions and the assessment measures up to that point. Given that users could not be followed up after they left the program, the last data point provided within the program represents a participant’s final data point. Analyses were also conducted based on the participants’ baseline and final assessment points (last assessment completed) to provide an overview of outcomes from the open-access program irrespective of the amount of treatment completed. For the majority of the sample, the final score was representative of approximately three sessions completed. Furthermore, a post-hoc power analysis revealed that the “completer” sample size for children (n=532) and adolescent (n=563) groups provided the power of 1.00 in detecting the within-subjects effect over four time points.

Given the large amount of missing data from participants who failed to provide data at all four assessment points, we also analyzed the full sample through latent growth curve modeling (LGCM) to confirm the findings of the completer analysis. LGCM is accepted as a suitable framework for use in the evaluation of efficacy in psychological interventions [22] and allows determination of whether the temporal trajectory from the baseline to Session 10 is significant, using data from all participants rather than just the treatment completers [22]. Time was specified as linear over the active treatment phase (baseline, Session 4, Session 7, and Session 10) in the growth models, and residual variances were held equal across time. Furthermore, models were fitted with a full-information maximum likelihood estimator using the Mplus 8 program [23]. We examined anxiety symptom trajectories separately for child and adolescent samples, in line with the repeated-measures ANOVA. For both the ANOVA and growth curve models, results were converted to standardized effect sizes (Cohen *d*).

The second method for analyzing changes in anxiety included utilization of the Reliable Change Index (RCI) [24]. RCI is a psychometric criterion that evaluates whether an individual participant changes sufficiently on a target measure (eg, CAS-8) over time (eg, from baseline to Session 4) and whether this change can be considered statistically significantly greater than the difference that might have been expected due to measurement error or unreliability [24]. In addition, RCI assesses whether the difference between two scores is more than a set level, determined by the product of the instrument’s SD and reliability [24]. Changes in scores can subsequently be categorized as “reliable improvement,” “no improvement,” or “reliable deterioration.” Given the lack of a control comparison condition in this study, RCI affords an opportunity to provide more rigorous analysis of the data and to illustrate statistically reliable change at the individual level for those participants who provided data at more than one time point. In this study, we calculated reliable change scores for each participant using the CAS-8 and presented the proportion of participants demonstrating reliable improvement, no improvement, or deterioration for each of the time contrasts (baseline to Session 4, baseline to Session 7, and baseline to Session 10) outlined above. Furthermore, RCI was estimated to be equivalent to a 4-point change for males and a 5-point change for females using reliability coefficients and gender-standardized norms for the CAS-8 based on Australian school-aged youth [18].

The third method of assessing change in anxiety was through examination of the proportion of individual youth cases crossing the clinical threshold [20] into recovery at a group level. The clinical threshold was deemed as a CAS-8 score ≥94th percentile (ie, *T*-score of ≥65) based on a large-scale community sample [18]. Of all participants, 51.32% (2271/4425; 632/1473 [42.91%] children and 1639/2952 [55.52%] adolescents) were categorized as being above the clinical threshold on the CAS-8 at enrollment. The remaining 48.68% (2154/4425) of the sample met criteria for “elevated” but not “clinical” levels of anxiety. We, therefore, also examined the proportion of youth crossing from the “elevated” threshold (84th percentile, *T*=60) to the “nonelevated” range. All analyses are presented separately for child and adolescent program users.

## Results

### Program Satisfaction

**Figure 2:**
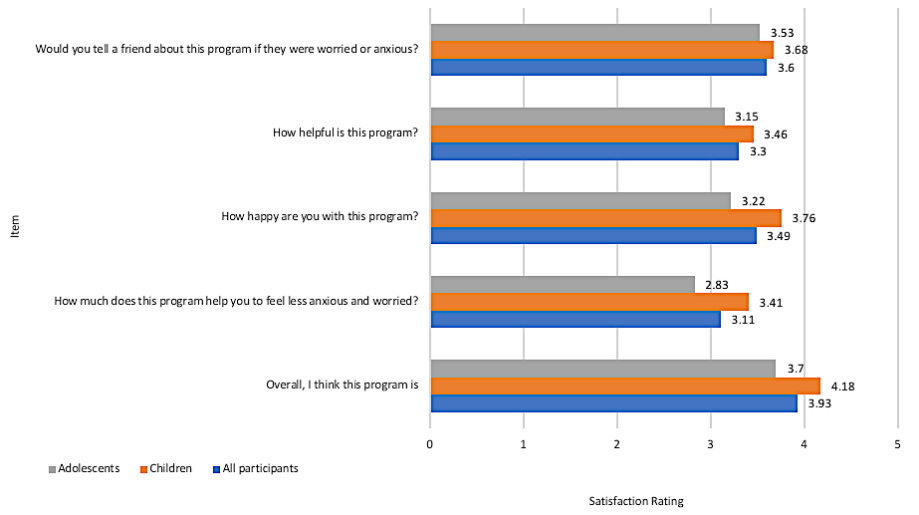
The mean satisfaction ratings for individual satisfaction items.

Using the last provided satisfaction scores for each participant, the mean total satisfaction rating was 17.72 (SD 5.16) out of a maximum 25. The mean satisfaction ratings for the individual items are provided in Figure 2 for all participants and across children and adolescents.

**Figure 3:**
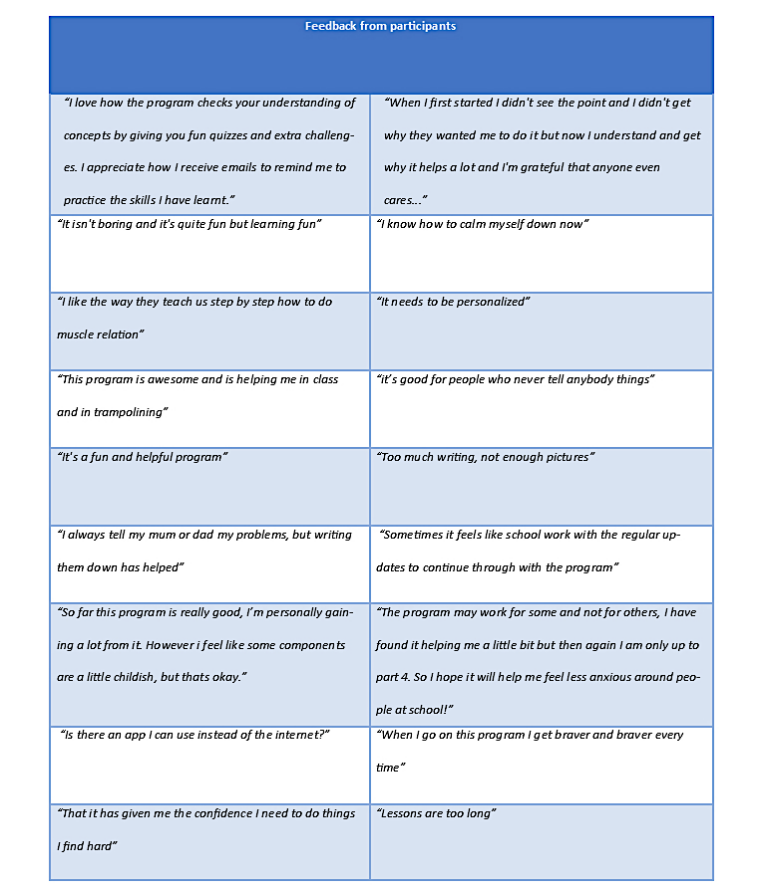
Feedback comments from participants.

In terms of the open feedback item, Figure 3 presents a list of comments provided by a snapshot of participants.

### Program Adherence

As indicated in Figure 1, only 24.75% (1095/4425) of the participants who demonstrated elevated anxiety at registration provided data at the second assessment point at Session 4 (and, thus, had completed at least three sessions). Participants who completed three or more sessions were younger (mean 11.90 years, SD 2.88) than those completing less than three sessions (mean 13.40 years, SD 2.89; *F*_1,4423_=252.68; *P*<.001). Furthermore, the former showed lower baseline anxiety severity (mean 14.86, SD 3.26), than the latter (mean 15.42, SD 3.40; *F*_1,4423_=26.02; *P*<.001). In addition, the former were more likely to be females (827/1341, 61.67%) than males (495/1341, 36.91%; *χ*^2^_1,4344_=22.4; *P*<.001) and other gender (19/1341, 1.41%) and were more likely to reside in major cities (741/1282, 57.80%) than in nonmetropolitan areas (541/1282, 42.20%; *χ*^2^_1,4184_=6.8; *P*=.009).

**Figure 4:**
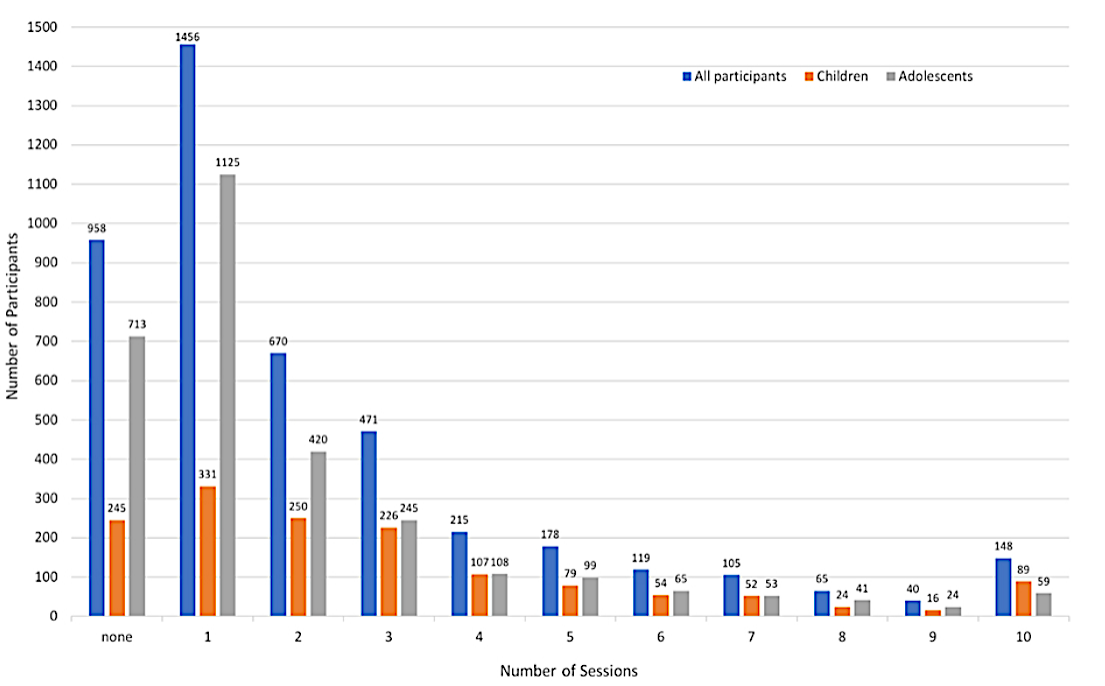
A visual summary of how many of the registered participants (N=4425) completed how many sessions of BRAVE Self-Help during the 20-week period (including participants who only provided one assessment point).

Of note, a large proportion of registered participants (958/4425, 21.65%) did not go on to complete the first session. A further 48.05% (2126/4425) of registered participants completed only one or two sessions of the program. However, 30.31% (1341/4425) participants went on to complete three or more sessions, with 1095 of these providing assessment data for at least two time points. The average number of sessions completed for all registered participants (including those who did not start the program) was 2.21 (SD 2.44). Figure 4 provides a visual summary of how many sessions were completed by all participants who had registered for the program, including a breakdown of sessions completed by children and adolescents.

For those participants who provided at least two data points (and, therefore, had completed at least three sessions), the average number of sessions completed was 5.69 (SD 2.35) out of 10. On average, child users completed 5.77 sessions (SD 2.45), while adolescent users completed 5.62 sessions (SD 2.25). A significant negative correlation was evident between the number of sessions completed and final CAS-8 scores (*r*=−.17; *P*=.003), such that higher session completion correlated with the lower final anxiety severity.

### Mean Changes in Anxiety

**Figure 5:**
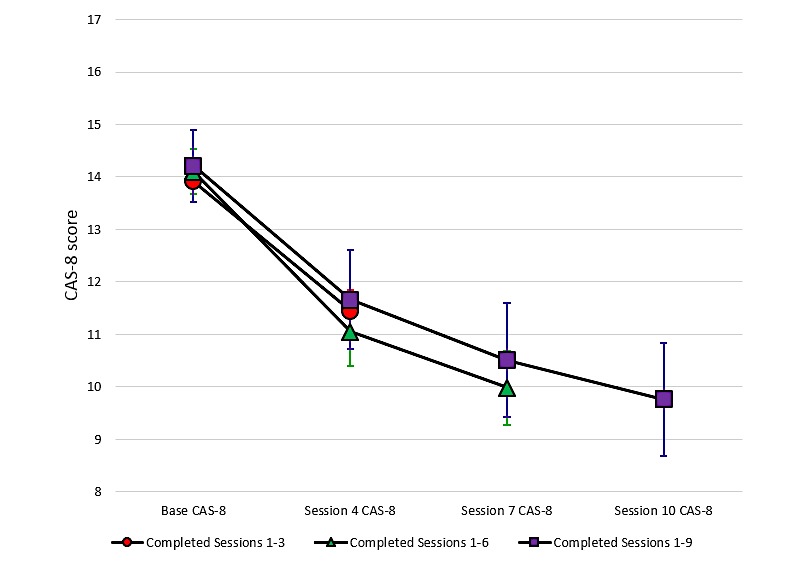
Changes in the mean anxiety scores according to the number of sessions completed for child program users. CAS-8: Children’s Anxiety Scale, 8-item.

**Figure 6:**
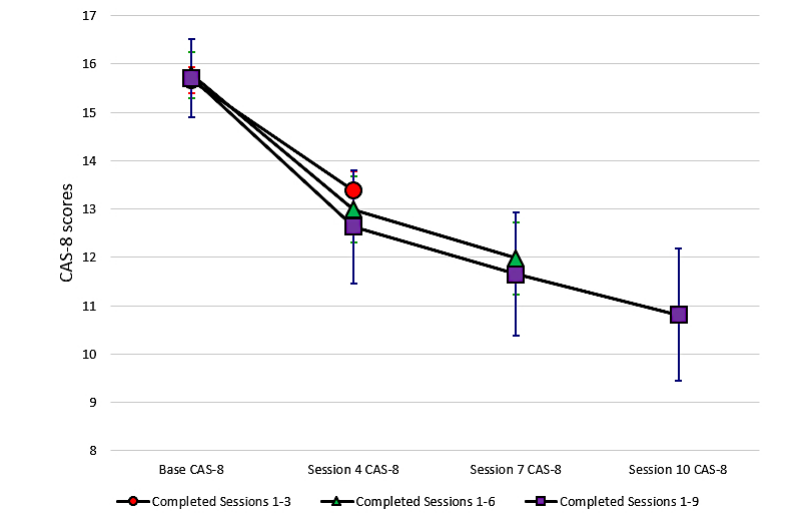
Changes in the mean anxiety scores according to the number of sessions completed for adolescent program users. CAS-8: Children’s Anxiety Scale, 8-item.

Changes in the CAS-8 score over time are provided for the child program participants in Figure 5 and adolescent program participants in Figure 6 (completer sample). These graphs plot the mean CAS-8 scores at the four time points for which anxiety was assessed and relate to those individuals who were retained in the program up to and including the session in which the assessment was completed. Thus, the number of participants present at each assessment point decreased as the number of completed sessions increased, as described above.

**Figure 7:**
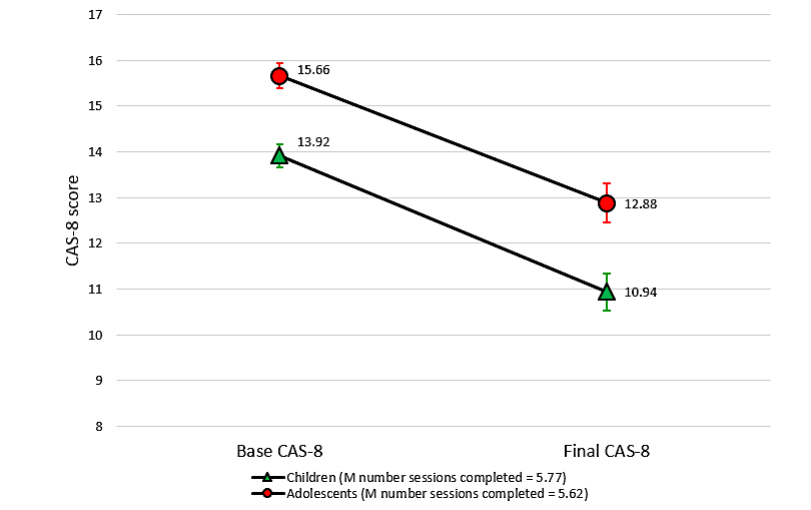
Changes in the mean anxiety scores from a user’s baseline to final CAS-8 score. CAS-8: Children’s Anxiety Scale, 8-item.

The mean CAS-8 scores over time and the results of ANOVAs and effect sizes are presented in Multimedia Appendix 1. We observed significant reductions in anxiety from the baseline to Session 4, baseline to Session 7, and baseline to Session 10 for both children and adolescents. For users who completed six or more sessions, we noted an average 4-point improvement in CAS-8 scores (Cohen *d*=0.88, children; Cohen *d*=0.81, adolescents), indicating a moderate to large effect size. The mean change was slightly less from the baseline to Session 4, although still significant, and was greater for those who completed the program up to the final Session 10 (see Multimedia Appendix 1). Figure 7 shows the change in CAS-8 scores for all participants from the baseline to their final data point before ending their engagement with the program. Results show that irrespective of the number of sessions completed, young people showed a statistically significant average decrease of around 3 points on the CAS-8 for both the child (Cohen *d*=0.66) and adolescent (Cohen *d*=0.65) programs.

The results for LGCMs confirm the findings of the completer sample and demonstrate that anxiety decreased significantly over time. Estimated means, SDs, and effect sizes for the CAS-8 in the child and adolescent samples are provided in Table 1. Linear growth curve models were estimated to evaluate the effect of time from the baseline to Session 10 on this anxiety outcome measure. For children, a statistically significant decrease was noted in anxiety from the baseline to Session 10 (*B*=−1.95; beta=−1.13; standard error [SE]=0.13; *P*<.001). In the adolescent sample, likewise, a statistically significant decrease was noted in anxiety from the baseline to Session 10 (*B*=−1.89; beta=−1.00; SE=0.13; *P*<0.001).

### Reliable Change

The proportion of child and adolescent users demonstrating statistically reliable (gender-adjusted) improvement, deterioration, or no change according to sessions completed is presented in Multimedia Appendix 2. The percentage of youth showing statistically reliable improvement increased as the number of sessions completed increased, with no significant differences between the proportions of children and adolescents showing change. Across the entire sample, for those who completed nine sessions (assessment completed at the beginning of Session 10), 54.6% (89/163) demonstrated statistically reliable improvement on the CAS-8, 40.5% (66/163) showed no statistically reliable change, and 4.9% (8/163) showed deterioration. For the entire sample, irrespective of the number of sessions completed, by their final recorded assessment, 35.62% (390/1095) showed reliable improvement in anxiety, 59.91% (656/1095) showed no statistically reliable change, and only 4.47% (49/1095) showed deterioration. Importantly, for child participants, only a very small proportion showed deterioration. Specifically, 4.32% (23/532), 3.63% (7/193), and 7.70% (7/91) of children showed deterioration after three, six, and nine sessions, respectively. This figure was even lower for adolescents, with only 3.20% (18/563), 4.88% (10/205), and 1.39% (1/72) showing deterioration after three, six, and nine sessions, respectively.

**Table 1:** Estimated Children Anxiety Scale, 8-item, means, SDs, and effect sizes from growth curve analyses.

Group	Baseline, mean (SD)	Session 4		Session 7		Session 10	
		Mean (SD)	Cohen *d*^a^	Mean (SD)	Cohen *d*^b^	Mean (SD)	Cohen *d*^c^
Children	14.09 (3.06)	11.55 (4.59)	0.60	10.24 (4.96)	0.82	9.57 (5.17)	0.86
Adolescents	15.83 (3.36)	13.51 (4.70)	0.60	12.42 (5.47)	0.74	11.53(5.77)	0.89

^a^Effect size from the baseline to Session 4.

^b^Effect size from the baseline to Session 7.

^c^Effect size from the baseline to Session 10.

### Proportion Crossing the Elevated and Clinical Thresholds

The proportion of child and adolescent users crossing the “elevated” and “clinical” thresholds at the different time points (and according to sessions completed) is provided in Multimedia Appendix 3. The change from elevated or clinical anxiety status into nonelevated anxiety status is an indicator of recovery. Similarly, the change from being above the clinical threshold to elevated (but not clinical) is an indicator of response (clinically meaningful improvement). The proportion of youth demonstrating recovery or response on these indicators increased as the number of sessions completed increased. For those who completed six sessions (Session 7 assessment), 53.0% (211/398) crossed from the “elevated” to “nonelevated” range (ie, demonstrated recovery); this increased slightly to 57.7% (94/163) for those who completed nine sessions. Across all users, 53.88% (590/1095) showed a reduction from the “elevated” anxiety range into the “nonelevated” range from the baseline to their final CAS-8 score before ending their engagement with the program, although a significantly greater proportion of children (325/532, 61.1%) than of adolescents (265/563, 47.1%) showed this change (*χ*^2^_1,1095_=21.64; *P*<.001).

For those who initially demonstrated “clinical” levels of anxiety, 59.1% (309/523) demonstrated an improvement into the “nonclinical” range by their final recorded score. We observed no differences in the proportion of children (134/217, 61.8%) and adolescents (175/306, 57.2%) achieving this level of improvement (*χ*^2^=1.09; *P*=.30). As is evident in Multimedia Appendix 3, of those who were initially in the “clinical range” of anxiety and who completed at least nine sessions of the program, 52 of 74 youth (26/37 children; 26/37 adolescents) no longer experienced clinical levels of anxiety. As demonstrated in Multimedia Appendix 4, of those participants demonstrating clinical anxiety at the baseline, 34 of 74 (17/37 children; 17/37 adolescents) reduced to the normal range of anxiety (recovered) and 18 of 74 (9/37 children; 9/37 adolescents) reduced to the elevated range (responded).

### Safety Alerts

The proportion of young people receiving email alerts for the presence of clinical-level anxiety was higher among adolescents than among children across all four time points and showed reduction over time for both groups. At the baseline (registration), 59.96% (1770/2952) of adolescents and 38.15% (562/1473) of children received email alerts, with this difference being statistically significant (*χ*^2^_1,4425_=187.5; *P*<.001). For adolescents, this number dropped to 39.8% (224/563) for those completing three sessions, 31.2% (64/205) for six sessions, and 29% (21/72) for nine sessions. For children, only 21.1% (112/532) received email alerts after completing three sessions, 14.5% (28/193) after six sessions, and 16% (15/91) after nine sessions.

## Discussion

### Study Objectives

This study reports on the feasibility and acceptability of a free, self-help iCBT intervention, offered nationally in Australia, and highlights the potential benefits, yet significant challenges evident, with this type of service delivery. The BRAVE Self-Help initiative was designed to provide an evidence-based intervention to anxious Australian children and adolescents while minimizing user burden and barriers to receipt of treatment (eg, cost, accessibility, stigma, and privacy). This study examined the feasibility and acceptability of this approach by evaluating satisfaction with, and adherence to, the program as well as changes in anxiety symptoms. Although there was no control comparison condition or trial methodology, the methodological approach to analysis was comprehensive, with data being collected on multiple occasions and the effects assessed using multiple methods of evaluating changes in anxiety. Furthermore, both satisfaction and adherence data provided further information about the acceptability of the open-access intervention.

### Intervention Acceptability

The BRAVE Self-Help program is extremely comprehensive as all specialist techniques are incorporated into the content and prominent examples and opportunities for skill rehearsal are integrated within and between sessions. In addition, the content is interactive, engaging, and age appropriate, and our previous research has demonstrated high consumer acceptability and satisfaction [10,11]. Thus, it was not surprising to find moderate to high satisfaction ratings reported by children and adolescents participating in the BRAVE Self-Help program. Slightly lower satisfaction ratings were reported by adolescents for all items, although the ratings remained moderate overall. As a group, participants were happy with the program, rated the overall program highly, and would refer the program to a friend if he or she experienced anxiety. Interestingly, satisfaction reported by users of this self-help version of the program was highly similar and in some cases, higher than that reported by youth participating in the therapist-assisted version of the program in our previous randomized controlled trials (RCTs; child program, mean satisfaction rating 3.6/5) [10] (adolescent program, mean satisfaction rating 3.53/5) [11]. Thus, satisfaction was not diminished when delivered as a self-help, open-access program with widespread dissemination.

Qualitative feedback indicated that many users were able to obtain benefits from the program, and potential improvements were noted by others. In particular, the intervention might benefit from accompanying app-based features, either for the entire program or for specific intervention components such as exposure. Furthermore, when delivered in this self-directed format, sessions might need further refinement to decrease the length, increase the use of videos and acceptable graphics, and reduce monitoring and reminder systems (unless requested, or demonstrated as being important in stimulating engagement). Interestingly, the need for sessions to be personalized was raised, despite the intervention being run as a self-help program without any professional contact. Thus, there are opportunities to implement innovative technology-based methods for achieving treatment personalization (eg, utilizing algorithms to present personalized treatment content or messages based on previous session responses) in such open-access, self-help online interventions.

Despite positive feedback and satisfaction with the program, there was a noticeable variation in the degree of program adherence across users. The fact that around 21% (958/4425) of participants did not go on to complete any of the sessions and a further 48% (2126/4425) completed only two sessions or less indicates that the program was either not acceptable or potentially not useful to a substantial proportion of people. These rates of session progression are not dissimilar to those found in other large-scale, open-access eHealth interventions such as MoodGym (in a sample of 82,159 participants, 63% completed no modules, 27% completed only 1 module, and 10% completed 2 or more modules out of 5 modules) [25] or even in face-to-face clinic service delivery contexts such as headspace (mean sessions attended 4.1; 49% of patients completing two or less sessions) [21]. Furthermore, in the only existing implementation trial for childhood anxiety, Jolstedt et al [15] reported an average adherence of 6 of 12 modules, with only half of the sample reaching module 4, despite the presence of therapist assistance and parent involvement.

In all likelihood, these results may suggest that services that are widely or publicly available attract users from varying contexts and backgrounds, who will subsequently engage very differently with the programs than those in strictly controlled RCTs. With respect to the BRAVE Self-Help initiative, given the significant change observed in users completing only three sessions, it is possible that several participants engaged in the program until they obtained the benefit they needed, which might have occurred early on in the program. For others, such as those who did not commence the program at all, it is possible that their expectations were not aligned with what the program had to offer and that this only became apparent after registering. For others still, it may be possible that a self-directed program with little guidance was simply not enough to sustain engagement. In this study, we were unable to determine the reasons for nonadherence, and this will be important for future studies. Nonetheless, while the program was acceptable, the low rates of adherence suggest that such approaches will not be sufficient for or acceptable to all.

### Changes in Anxiety

The results of this study demonstrate that self-reported anxiety decreased significantly over time, with effects being greater as the number of sessions completed by youth increased. These findings were confirmed through the completer analyses (N=1095) and growth curve modeling, which utilized all eligible participants registering for the program (N=4425). Improvements were evident for both children and adolescents, although adolescents showed slightly less improvement on some outcome indicators. Based on the assessment point at the beginning of Session 4, results showed that around 43% of children and around one-third of adolescents had recovered (no longer experienced elevated anxiety). For those with clinical levels of anxiety, over half (57%) of children and just under half (45%) of adolescents were no longer in the clinical range after completing three sessions (ie, showed response). Importantly, even greater reductions were evident for child and adolescent users who completed more sessions, such that of those who completed nine sessions, over two-thirds (70%) were no longer in the clinical range (ie, showed response) and almost half (47%) no longer demonstrated elevated anxiety (ie, showed recovery). Furthermore, of all participants who completed nine sessions, around half (54%) achieved statistically reliable change as indicated by RCI. Thus, we observed substantial reductions in anxiety across multiple measures among users of a freely available, evidence-based online intervention.

Although not directly comparable, as diagnostic status was not determined by clinical interview in this study, the results can be compared with those of the previous RCTs of the therapist-assisted BRAVE-ONLINE program. The demonstrated effect sizes for reductions in anxiety from the baseline to Session 9 in this study (Cohen *d*=0.83-1.01) are similar to the effect sizes observed in previous RCTs (Cohen *d*=0.91-1.23 for the full Spence Children’s Anxiety Scale) [10,11]. Furthermore, the proportion of young people with clinical levels of anxiety at the baseline who no longer reported anxiety (47%) after participating in BRAVE Self-Help is similar to the proportion of youth no longer meeting diagnostic criteria after 12 weeks in the above-mentioned RCTs (between 30% and 37%). However, it is important to note that different (briefer) outcome measures were utilized in this study compared with the comprehensive diagnostic assessments obtained in RCTs. Thus the results of this study are not directly comparable and should be interpreted with caution.

The effect sizes for BRAVE Self-Help presented in this study are somewhat smaller than those of a pilot implementation study (N=20) conducted very recently by Jolstedt et al [15]. In their study, the authors reported an effect size of Cohen *d*=1.22 on the full Spence Children’s Anxiety Scale from pre- to posttreatment, although it should be noted that this intervention was (1) delivered with substantial therapist support (regular asynchronous contact through messages, comments on activities, and phone calls), (2) required both parents and children to complete sessions before subsequent sessions were unlocked, and (3) encouraged families to log in at least twice a week to respond to therapists [15]. Thus, BRAVE Self-Help is a lower-intensity intervention than the one described by Jolstedt et al, yet with only somewhat lesser effects.

### Implications

It is often argued that low-intensity interventions such as iCBT programs are only effective with professional support. However, the results of this study demonstrate that self-help online interventions may be effective for many young people if they complete at least three sessions of the program. Indeed, the improvements demonstrated by participants in this open-access, self-help program are significant and reveal that a meaningful change is feasible without therapist support. The finding that a substantial proportion of young people can achieve clinically meaningful improvements through such an intervention in a self-help format has significant implications for models of service delivery. In addition, substantial therapist time and cost savings may be afforded by such self-help interventions, contributing to an increase in the overall efficiency of youth mental health services. Furthermore, providing evidence-based services via online self-help may reach more young people at a far lower cost than face-to-face service delivery models. It would seem that if young people who would benefit from self-help programs are accurately identified, more costly resources (eg, face-to-face therapist sessions) could be reserved for those young people who need them the most.

Another implication of the findings relates to the treatment dose or magnitude of change in early sessions. Specifically, it is worth noting that the highest magnitude of change was evident following the completion of the first six sessions. Thus, the results of this study demonstrate that even a small dose of self-help treatment may be effective for some young people and would perhaps bring about a change equivalent to that from an extended program. These findings are also consistent with those of Chu et al [26] who demonstrated a nonlinear symptom trajectory for youth engaging in face-to-face CBT; in fact, they reported that participants tended to show a rapid response over the first six sessions, with changes tending to taper off thereafter. Therefore, if used as the first step in a stepped-care service provision model, users may potentially require only six sessions or fewer. This makes sense given that the first six sessions of the program contain the specialist CBT skills and skill rehearsal, with Sessions 7-10 targeting practice with exposure tasks and relapse prevention. Therefore, not only do the results of this study support the benefits of online self-help interventions but also assist in identifying the potential trajectory of a symptom change or the “ideal dose” of such low-intensity programs.

### Limitations and Future Research

This open dissemination study has some design limitations. There was no control group against which to determine whether changes simply reflect spontaneous recovery, regression to the mean, or nonspecific intervention effects. In addition, there was no initial interview to confirm the clinical diagnosis, instead a single informant and a single measure was relied upon. Although this measure is unable to provide a comprehensive overview of anxious symptomatology type and intensity, it does allow for examination of the magnitude of anxiety symptom change, which was the objective of this study. Furthermore, it was necessary to minimize participant burden in an open trial of this nature where participation was completely voluntary and self-sought.

We also note that some analyses in this study were limited to those individuals who completed at least two assessment points and, thus, had completed at least three program sessions. While this setting provides a fair evaluation of the outcome for those who completed at least a proportion of the program, it does not consider those who initially enrolled and decided not to continue at all with the program. Perhaps, such young people were not ready to participate in active treatment or they might require alternative treatment modalities. In fact, in this study, those who completed less than three sessions were more likely to reside in non-metropolitan areas, were older and had a higher anxiety severity. It is possible that low-intensity iCBT programs may be more accessible or preferred by youth who live in major cities. Also, younger children may be more likely than adolescents to complete iCBT programs with parental assistance, which may bring about higher adherence to the program. Furthermore, the finding that youth with lower levels of anxiety adhered more to the program is consistent with the objectives of low-intensity iCBT interventions and supports the notion that iCBT for youth could be useful as a first step in intervention.

In terms of sample representativeness, all participants in this study demonstrated elevated anxiety > 84th percentile on the CAS-8. Thus, all participants were experiencing anxiety at a level that was interfering with their lives; however, only half of the participants demonstrated “clinical” levels of anxiety (>94th percentile). Thus, the sample was somewhat less severe than those examined in previous RCTs of iCBT for child anxiety [10,11]. Participants in this study included both children and adolescents, with similar mean ages to other trials on child anxiety. However, there was a slightly higher proportion of females (61%) in this study than in previous trials that had a more even gender distribution [10,11]. Finally, although the BRAVE Program does offer parent modules, in this open-access, anonymous delivery model, we were unable to link parent and child accounts and, thus, could not determine the impact of parental involvement.

Despite its limitations, the findings of this study are encouraging and certainly justify further research in an RCT or similar design to confirm the effectiveness of this type of intervention. Historically, there has been a lack of formalized approaches to the dissemination of iCBT interventions [14], yet there are some recent design recommendations that may be appropriate. To address the challenges of conducting controlled trials in real-world implementation settings, it may be necessary to conduct a stepped-wedge cluster design, which is increasingly recommended for evaluation of service delivery interventions [27]. Such research should also consider the inclusion of clinician-, parent-, or teacher-informant reports to provide a more comprehensive diagnostic information. Furthermore, future research should incorporate more frequent assessments of anxiety to enable the examination of response trajectories according to baseline individual and clinical factors. Such analyses will assist in defining the ideal treatment dose as well as predictors of nonresponse and have the potential to provide evidence that could inform the design and implementation of “stepped-care” approaches. Finally, there is a pressing need for health economic evaluations to determine the relative cost and health benefits of online, self-help approaches compared with therapist-supported, online programs and face-to-face services.

### Conclusion

This online, self-help program for anxiety has the capacity to reach greater numbers of young people compared with programs that require therapist contact. The results of this open trial demonstrate moderate to high program acceptability when delivered in this way and show its potential feasibility in bringing about clinically and statistically meaningful reductions in anxiety for children and adolescents. Greater reductions were evident for those who completed more sessions, although significant improvements were most evident in the first six sessions. Overall, self-help iCBT is a potentially feasible and acceptable approach for delivering evidence-based interventions through a public health delivery model.

## Appendix

Multimedia Appendix 1 A summary of analysis of variance (ANOVA) and effect sizes.

Multimedia Appendix 2 A reliable change over time.

Multimedia Appendix 3 The proportion of participants crossing the "elevated" and "clinical" thresholds.

Multimedia Appendix 4 The proportion of clinical participants moving from "clinical" to "elevated" and "normal" anxiety levels.

## Abbreviations

ANOVA: analysis of variance
CAS-8: Children’s Anxiety Scale, 8-item
CBT: cognitive behavioral therapy
iCBT: internet-based cognitive behavioral therapy
LGCM: latent growth curve modeling
RCI: Reliable Change Index
RCT: randomized controlled trials
UQ: University of Queensland
