# Supplementary material for: Large-Scale Dissemination of Internet-Based Cognitive Behavioral Therapy for Youth Anxiety: Feasibility and Acceptability Study
Source: J Med Internet Res. 2018 Jul 4;20(7):e234. doi: 10.2196/jmir.9211 (PMC6053603; doi:10.2196/jmir.9211)
Supplement: Multimedia Appendix 1 [file jmir_v20i7e234_app1.pdf]

Multimedia Appendix 1. Summary of means, ANOVAs and effect sizes

**Analysis**<br><br>(Change in CAS between two time points)	**Baseline CAS**<br><br>**M (SD)**	**Session 4 CAS**<br><br>**M (SD)**	**Session 7 CAS**<br><br>**M (SD)**	**Session 10 CAS**<br><br>**M (SD)**	**Final CAS**<br><br>**M (SD)**	**Statistics**
Child Program						
Baseline to Final CAS	13.92 (2.96)	-	-	-	10.94 (4.82)	F(1,531) = 209.71, p<.001, n^2 = .28, Cohen’s d = .66
Baseline to Session 4	13.92 (2.96)	11.45 (4.59)	-	-	-	F(1,531) = 169.20, p<.001, n^2 = .24, Cohen’s d = .59
Baseline-Session 4-Session 7	14.10 (3.03)	11.05 (4.60)	9.98 (4.97)	-	-	F(2,191) = 73.82, p<.001, n^2 = .44, Cohen’s d = .88
Baseline-Session 4-Session 7-Session 10	14.21 (3.29)	11.66 (4.50)	10.51 (5.24)	9.76 (5.20)	-	F(3,88) = 19.95, p<.001, n^2 = .41, Cohen’s d = .83
Adolescent Program						
Baseline to Final CAS	15.66 (3.30)	-	-	-	12.88 (5.05)	F(1,562) = 211.19, p<.001, n^2 = .27, Cohen’s d = .65
Baseline to Session 4	15.66 (3.30)	13.38 (4.70)	-	-	-	F(1,562) = 179.90, p<.001, n^2 = .24, Cohen’s d = .59
Baseline-Session 4-Session 7	15.77 (3.48)	12.99 (4.93)	11.98 (5.48)	-	-	F(2,203) = 62.22, p<.001, n^2 = .38, Cohen’s d = .81

Baseline-Session 4-Session 7-Session 10	15.71 (3.45)	12.63 (4.98)	11.65 (5.40)	10.81 (5.81)	-	F(3,69) = 21.60, p<.001, n^2 = .48, Cohen's d = 1.01

Note. Final CAS = the last CAS assessment the user completed
