# Supplementary material for: Large-Scale Dissemination of Internet-Based Cognitive Behavioral Therapy for Youth Anxiety: Feasibility and Acceptability Study
Source: J Med Internet Res. 2018 Jul 4;20(7):e234. doi: 10.2196/jmir.9211 (PMC6053603; doi:10.2196/jmir.9211)
Supplement: Multimedia Appendix 2 [file jmir_v20i7e234_app2.pdf]

Multimedia Appendix 2. Proportion of participants showing reliable improvement, no change or deterioration in anxiety, according to program, number of sessions and data collection point.

| Number of Sessions completed | Data collection point | N | No statistically reliable change[a] N (%) | Reliable Improvement[b] N (%) | Showed Deterioration[c] N (%) |
|---|---|---|---|---|---|
| Child Program | | | | | |
| Completed 3 sessions | CAS 4 | 532 | 342 (64.28) | 167 (31.39) | 23 (4.32) |
| Completed 6 sessions | CAS 7 | 193 | 94 (48.70) | 92 (47.67) | 7 (3.63) |
| Completed 9 sessions | CAS 10 | 91 | 36 (39.56) | 48 (52.74) | 7 (7.70) |
| Adolescent Program | | | | | |
| Completed 3 sessions | CAS 4 | 563 | 389 (69.09) | 156 (27.71) | 18 (3.20) |
| Completed 6 sessions | CAS 7 | 205 | 108 (52.68) | 87 (42.44) | 10 (4.88) |
| Completed 9 sessions | CAS 10 | 72 | 30 (41.67) | 41 (56.94) | 1 (1.39) |

a = any changes made were less than 4 points (males) or 5 points (females) on the CAS-8
b = showed a reduction of 4 points or more (males) or 5 points or more (females) on the CAS-8
c = showed an increase of 4 points or more (males) or 5 points or more (females) on the CAS-8
