# Supplementary material for: Large-Scale Dissemination of Internet-Based Cognitive Behavioral Therapy for Youth Anxiety: Feasibility and Acceptability Study
Source: J Med Internet Res. 2018 Jul 4;20(7):e234. doi: 10.2196/jmir.9211 (PMC6053603; doi:10.2196/jmir.9211)
Supplement: Multimedia Appendix 3 [file jmir_v20i7e234_app3.pdf]

Multimedia Appendix 3. Proportion of participants crossing the elevated and clinical threshold according to program, number of sessions and data collection point.

		Participants initially elevated			Participants initially clinical		
Number of Sessions completed	Data point	Sample N	Remained Elevated (T-score ≥60) N (%)	Crossed Elevated Threshold (T-score <60)[a] N (%)	N	Remained Clinical (T-score ≥65) N (%)	Crossed Clinical Threshold (T-score <65)[b] N (%)
Child Program							
Completed 3 sessions	CAS 4	532	303 (56.95)	229 (43.05)[c]	217	83 (38.25)	134 (61.75)[d]
Completed 6 sessions	CAS 7	193	85 (44.04)	108 (55.96)	85	25 (29.41)	60 (70.59)[d]
Completed 9 sessions	CAS 10	91	36 (39.56)	55 (60.44)	37	11 (29.73)	26 (70.27)
Adolescent Program							
Completed 3 sessions	CAS 4	563	387 (68.74)	176 (31.26)	306	131 (42.81)	175 (57.19)
Completed 6 sessions	CAS 7	205	102 (49.76)	103 (50.24)	106	48 (45.28)	58 (54.72)
Completed 9 sessions	CAS 10	72	33 (45.83)	39 (54.17)	37	11 (29.73)	26 (70.27)

[a] Crossed elevated threshold into normal levels of anxiety
[b] Crossed clinical threshold into either elevated or normal levels of anxiety
[c] indicates a significantly higher proportion crossing elevated/clinical threshold compared to adolescents at $p<.001$
[d] indicates at $p<.05$
