# Supplementary material for: Large-Scale Dissemination of Internet-Based Cognitive Behavioral Therapy for Youth Anxiety: Feasibility and Acceptability Study
Source: J Med Internet Res. 2018 Jul 4;20(7):e234. doi: 10.2196/jmir.9211 (PMC6053603; doi:10.2196/jmir.9211)
Supplement: Multimedia Appendix 4 [file jmir_v20i7e234_app4.pdf]

Multimedia Appendix 4. Breakdown of participants moving from ‘clinical’ to ‘elevated’ and ‘normal’ anxiety levels according to program, number of sessions and data collection point.

Number of Sessions completed	Data collection point	N	Remained Clinical (T-score ≥65) N (%)	Reduced to Elevated (T-score <65) N (%)	Reduced to Normal (T-score <60) N (%)
Child Program					
Completed 3 sessions	CAS 4	217	92 (42.39)	54 (24.88)	71 (32.73)
Completed 6 sessions	CAS 7	85	25 (29.41)	29 (34.12)	31 (36.47)
Completed 9 sessions	CAS 10	37	11 (29.73)	9 (21.62)	17 (45.95)
Adolescent Program					
Completed 3 sessions	CAS 4	306	169 (55.23)	79 (25.82)	58 (18.95)
Completed 6 sessions	CAS 7	106	48 (45.28)	21 (19.81)	37 (34.91)
Completed 9 sessions	CAS 10	37	11 (29.73)	9 (21.62)	17 (45.95)
